# Future ocean biomass losses may widen socioeconomic equity gaps

**DOI:** 10.1038/s41467-020-15708-9

**Published:** 2020-05-06

**Authors:** Daniel G. Boyce, Heike K. Lotze, Derek P. Tittensor, David A. Carozza, Boris Worm

**Affiliations:** 1 Ocean Frontier Institute, Dalhousie University, Halifax, NS Canada B3H 4R2; 2 Department of Biology, Dalhousie University, Halifax, NS Canada B3H 4R2; 3 Département de Mathématiques, Université du Québec à Montreal, Montreal, QC Canada H2X 3Y7

**Keywords:** Climate-change ecology, Ecological modelling, Ecosystem services, Marine biology

## Abstract

Future climate impacts and their consequences are increasingly being explored using multi-model ensembles that average across individual model projections. Here we develop a statistical framework that integrates projections from coupled ecosystem and earth-system models to evaluate significance and uncertainty in marine animal biomass changes over the 21^st^ century in relation to socioeconomic indicators at national to global scales. Significant biomass changes are projected in 40%–57% of the global ocean, with 68%–84% of these areas exhibiting declining trends under low and high emission scenarios, respectively. Given unabated emissions, maritime nations with poor socioeconomic statuses such as low nutrition, wealth, and ocean health will experience the greatest projected losses. These findings suggest that climate-driven biomass changes will widen existing equity gaps and disproportionally affect populations that contributed least to global CO_2_ emissions. However, our analysis also suggests that such deleterious outcomes are largely preventable by achieving negative emissions (RCP 2.6).

## Abstract (web summary)

Numerous marine ecosystem models are used to project animal biomass over time but integrating them can be challenging. Here the authors develop a test for statistical significance in multi-model ensemble trends, and thus relate future biomass trends to current patterns of ecological and socioeconomic status.

## Introduction

Understanding future changes in ocean health and service provision will be critical to achieving sustainable development while addressing socioeconomic inequality and conflicts over marine resources over the coming century^1^. Projections of future change under differing scenarios are crucial to help build such understanding. Global climate models (GCMs) resolve physical processes to make such projections for the climate system, and earth system models (ESMs) build on GCMs to also resolve biogeochemical processes^2^. Most recently, through the coupling of ESMs to global marine ecosystem models (MEMs), it is now possible to project how marine life, from microbes to top predators, will change under different greenhouse gas emissions, ocean warming, and fishery exploitation scenarios^3–5^. Such projections are increasingly being included in policy documents such as the Intergovernmental Panel on Climate Change Reports^6–8^ and used by international organizations such as the Food and Agricultural Organization of the United Nations (FAO) and the Intergovernmental Science-Policy Platform on Biodiversity and Ecosystem Services (IPBES), and inform decision-makers of how climate-driven ecological changes may affect biodiversity, food production and human well-being^9,10^. Such projections are also beginning to be incorporated into applied ocean management settings^9,11^.

Despite increasing reliance on such coupled models, the variability among individual projections, possibly due to differences in the structure, assumptions, and processes of the underlying models, can be a barrier to the effective interpretation and implementation of findings^12–16^. Projections of change from single models often deviate from and sometimes conflict with others^17,18^, prompting researchers to combine projections into multi-model ensemble averages (MMEAs). This MMEA approach is most commonly implemented as a “model democracy” considering all projections to be equally plausible and has been widely adopted as a more reliable alternative to individual projections^3,5,6,12,19,20^. Following the growing use of MMEAs, there have lately been calls to adopt alternative statistical approaches better suited for evaluating projection uncertainty^21^, exploring causation^22^, and increasing the statistical rigor of MMEA analyses^14^.

Combining ensemble projections using methods that allow statistical hypothesis testing would be a logical step toward increasing the rigor of MMEA analyses. While statistical hypothesis testing is a “gold standard” for distinguishing signals of change from other sources of variation^23^, and a requirement of most peer-review processes, it has rarely been implemented in MMEA analyses. The non-independence of models and the potential for extreme projections to dominate an ensemble average can bias the MMEA results in ways not yet fully understood^14,24^. Given the often-conflicting and uncertain nature of individual model projections, especially at high latitudes^5,17,20^, and the increasing extent to which projected changes are being used in applied settings^6,7,9,10^, the inability to resolve the significance and uncertainty of MMEA trends is a critical knowledge gap. Filling this gap, via hypothesis testing, would build confidence in the reliability of ensemble trends and may facilitate a greater understanding of how climate change impacts are related to the broader dimensions of socio-economic development, which is thus far largely unresolved. For instance, projections of marine ecosystem responses to climate change could be used to explore feasibility pathways towards meeting several of the sustainable development goals (SDGs), including those aimed at reducing hunger (SDG2), improving health, well-being (SDG3), and economic inequalities (SDG10), and avoiding adverse ecosystem effects due to climate change (SDG14).

Here, we address these knowledge gaps by estimating the rates of climate-driven ensemble-averaged animal biomass changes and their statistical significance over the 21st century and then relating these future biomass changes to present-day indicators of fisheries productivity, human stressors, and socioeconomic status (SES). Ensemble-averaged biomass changes and their significance were estimated using longitudinal models (also known as mixed-effects or hierarchical models). Longitudinal models are used in disciplines such as the health sciences^25–27^, psychology, finance, ecology^28^, and fisheries^29^ to understand shared associations that are manifest as multiple individual time-series. For example, longitudinal models have been used in epidemiological studies to statistically account for confounding differences in time-series of demographic health metrics, including body mass^25^, blood pressure^27^, and diabetes^26^ that may be due to variation between individuals, study methodologies, populations, and other factors, to enable robust conclusions about associations that operate globally. The approach is ideally suited to ensemble projections, where many time-series derived from models with potentially different architectures are used to describe an unknown—but shared—climate response through time. Despite their widespread application in other disciplines, longitudinal models remain thus far unused in ensemble climate forecasting.

We obtained annual time series of projected unfished marine animal biomass on a global 1 × 1° grid from the Fisheries, and Marine Ecosystems Model Inter-comparison Project (Fish-MIP^3,30^), which includes six global MEMs forced with the standardized output of two ESMs under two emission scenarios (Representative Concentration Pathways; RCP2.6 and RCP8.5). While the MEMs have very different underlying structures and assumptions (see Methods), the validity of their projections have been extensively validated against empirical observations^5,31,32^. Notwithstanding their increasing application and validation, the skill of MEM projections is not yet suitably high to warrant their use in applied ocean management at the requisite taxonomic and spatial scales (see Supplementary Discussion).

**Fig. 1:**
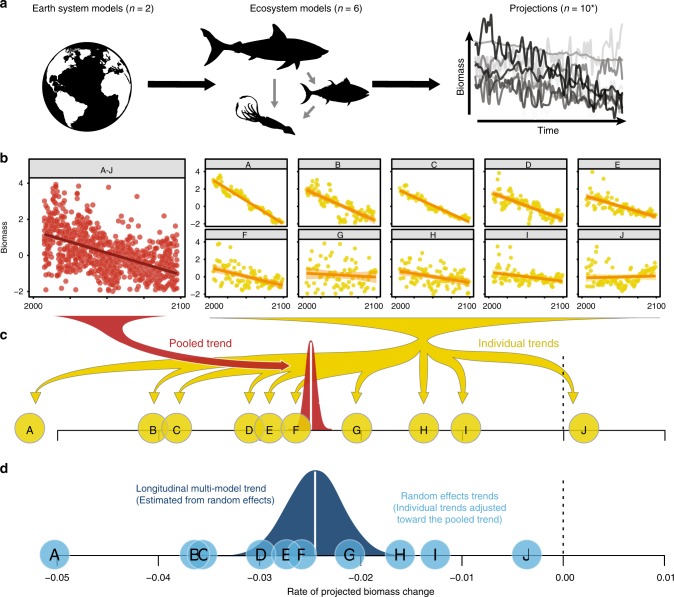
Projecting ecological change. **a** Projections of marine animal biomass under different emission scenarios are obtained by coupling the standardized output from two earth system models to six global marine ecosystem models. **b** Biomass trends (lines) are estimated from model projections (points) individually (yellow) and collectively in a single pooled sample (red). **c** Individual time trends (yellow shaded circles) are adjusted toward the pooled trend (red shaded density) according to their distance from it and their variability; more variable trends that are far from the pooled trend are adjusted the most (e.g., J). **d** Adjusted individual trends or random effects predictions (blue points) are used to derive the multi-model longitudinal biomass trend, and its statistical uncertainty (blue shaded density). The overall trend distribution often has a similar mean but a significantly larger variance than the pooled or individual trends.

For each emissions scenario and within each 1° grid cell, a longitudinal model was used to estimate the long-term trend in marine animal biomass ($\beta _t$; % yr^−1^) and its uncertainty (standard error; $\sigma _t$; % yr^−1^) while accounting for temporal autocorrelation. While examining the trends individually (yellow in Fig. 1b, c) would prohibit the evaluation of any shared pattern of biomass change across the ensembles, combining the projections to estimate a single “pooled” trend (red in Fig. 1b, c) would introduce bias due to the possibly correlated projections (collinearity^33^). The longitudinal approach optimizes the trade-off between these two approaches. The individual trends (yellow circles in Fig. 1c) are adjusted toward the pooled trend (white vertical line in Fig. 1c), with those that are less probable—e.g., they contain less data, have a high variance, and are far from the pooled trend (e.g., J in Fig. 1b, c) being adjusted to the greatest extent. The resulting estimates of change, or random effects predictions (blue shaded circles in Fig. 1d), are then used to estimate the multi-model longitudinal rate of change (white vertical line in Fig. 1d), along with its statistical uncertainty (blue density in Fig. 1d), and significance. Our analysis did not include different ecosystem model initializations or fishing scenarios. Therefore, the overall projection uncertainty estimated by the longitudinal models primarily relates to the structure and parameterization of the ecosystem models, rather than to initialization or scenario factors^15,16^, although these factors could be included in future analyses. See the methods section and refs. ^28,34^ for further details.

After analyzing the geographic patterns of ensemble biomass trends and their significance, we explore how these changes relate to socioeconomic indicators at national to global scales. Specifically, we compare marine biomass changes to indicators of fisheries productivity, human stressors, and SES to explore how impacts may affect different maritime states and their prospects for sustainable development. The longitudinal approach allows for the statistical uncertainty of the multi-model longitudinal trends to be carried forward through our analyses, thus building robustness. This work complements previous Fish-MIP studies^5,20^ by using a statistical approach that estimates the significance of ensemble trends in biomass, and by evaluating the broader socioeconomic implications.

## Results

### Global patterns of multi-model ensemble animal biomass

**Fig. 2:**
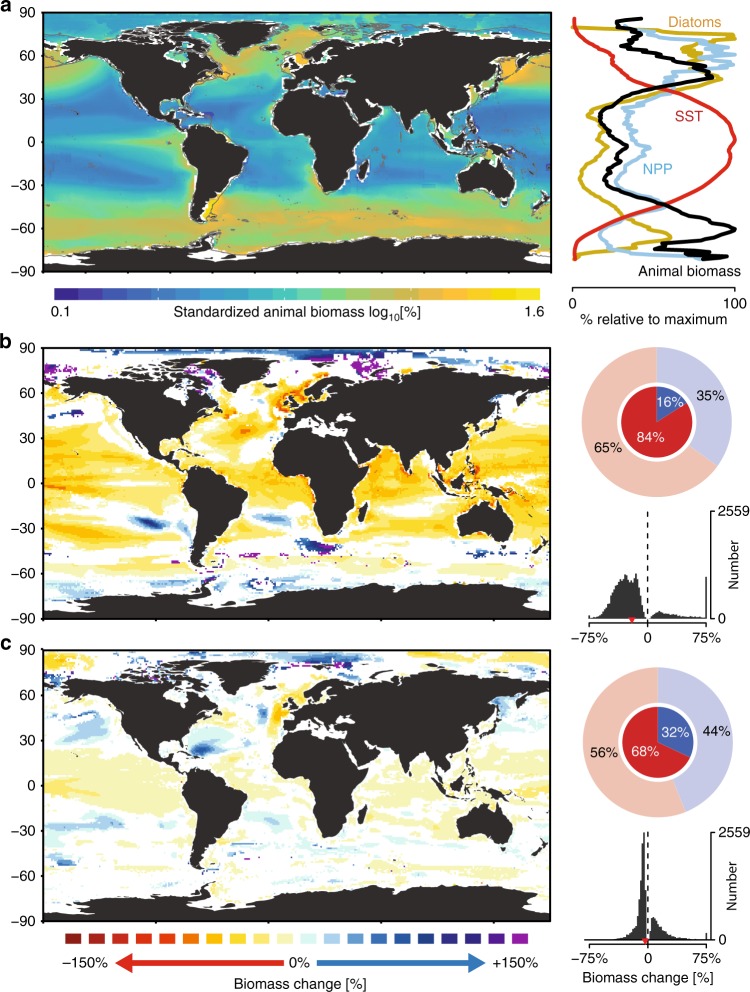
Global patterns of marine animal biomass and projected change. **a** Average standardized biomass of marine animals from multi-model projections for the contemporary period (2006–2016). Left: Map depicts animal biomass density in each grid cell, relative to the global maximum and normalized. Gray lines depict the 200 m isobath. Right: graph shows the latitudinal variation in present-day animal biomass (black), SST (red), NPP (blue), and diatom frequency (yellow). **b**, **c** Maps of projected future change in animal biomass between 2006 and 2100, relative to the reference period (2006–2016) under a worst-case scenario RCP8.5 (**b**) and strong mitigation scenario RCP2.6 (**c**). White depicts grid cells containing non-significant trends (*p* > 0.05) or containing insufficient data for analysis. Circular histograms depict the proportion of grid cells where analyses were possible that contained increasing (blue) or declining (red) changes. Inner opaque shading depicts changes that were statistically significant (*p* < 0.05), and outer shading depicts those that were both statistically significant and non-significant. Histograms show the distribution of all statistically significant predicted changes per grid cell with global means denoted as red arrows. Projected changes in **b**, **c** were estimated using longitudinal models. Data sources are listed in Table 1.

To evaluate present-day patterns of marine animal biomass, we calculated the multi-model ensemble mean biomass within each grid cell between 2006 and 2016 in standardized units of percentage of the global maximum (%; Fig. 2a). Peak biomass levels emerged in most upwelling regions, and at high temperate latitudes (~50–60°N and °S). The lowest animal biomass concentrations were observed at lower latitudes (<30°N and °S), particularly in the oligotrophic gyres. Average biomass was positively related to latitudinal gradients in average net primary production (NPP) and negatively related to gradients in sea surface temperature (SST), especially between ~50°N and °S, but less so at higher latitudes (Fig. 2a). Here, the prevalence of diatoms was often elevated, potentially increasing the fraction of NPP transferred to consumers rather than to the microbial loop, leading to higher animal biomass than would be expected from NPP or SST alone^4^. A linear regression model containing all three variables explained substantially more of the spatial variation in animal biomass (78%) than did any single variable (17–51%). These findings suggest that observed biomass patterns are explained by both biogeochemical (SST and NPP) and ecological variables (species composition) and highlight the importance of accurately incorporating microbial food chain dynamics and size-based predation^35^ as do most of the MEMs used here^3^. As we omitted cells containing <3 projections, biomass patterns could not be examined in many nearshore locations.

### Projected future changes in marine animal biomass

Climate change scenarios had a large effect on projected biomass trends. Under a worst-case scenario (RCP8.5, Fig. 2b), 84% of statistically significant trends (*p* < 0.05) projected a decline in animal biomass over the 21st century, with a global median change of −22%. Rapid biomass declines were projected across most ocean areas (60°S to 60°N) but were particularly pronounced in the North Atlantic Ocean. Under a strong mitigation scenario (RCP2.6, Fig. 2c), 68% of significant trends exhibited declining biomass, with a global median change of −4.8%. Despite the overall prevalence of negative trends, some large biomass increases (>75%) were projected, particularly in the high Arctic Oceans.

Our analysis suggests that statistically significant biomass changes between 2006 and 2100 will occur in 40% (RCP2.6) or 57% (RCP8.5) of the global ocean, respectively (Fig. 2b, c). For the remaining cells, the signal of biomass change was not separable from the background variability. The estimates of biomass change and their uncertainty were different from those obtained using multi-model ensemble averaging (see Supplementary Methods for details). When present, differences between longitudinal vs. MMEA trends tended to be large and driven by trends identified by the longitudinal models as non-significant. By using longitudinal models, these highly uncertain estimates can be identified and removed (Fig. 2b, c), or statistically accounted for in subsequent analyses.

### Relating future biomass changes to SES

To explore the consequences of projected biomass changes within a broader ecological and societal context, we related them to present-day geographical patterns of fisheries productivity, human stressors and indicators of SES. Indicators of fisheries productivity included annual reported and estimated commercial fishery landings, as well as illegal and unreported fishing activity (Table 1, Supplementary Table 1). Stressors included both multivariate indices of cumulative human impacts^36^, as well as individual stressors such as pollution, for example. SES indicators assessed development and nutritional status, social condition, ecological health, and climate change vulnerability of 106 maritime states (see Methods section, Table 1, and Supplementary Table 1 for a complete list of indicators). The SES indicators were split into those for which higher scores denoted improved condition (states), and those where increasing scores indicated reduced condition (pressures). Except for officially reported FAO fishery landings, which are made available at the scale of FAO statistical areas (*n* = 18), all spatial relationships between projected biomass changes and fisheries and human stressors were tested at the scale of marine ecoregions^37^, while SES indicators were examined at the scale of individual states’ exclusive economic zones (EEZs).

**Table 1:** Data sources.

Index	Category	Authority	Units	Resolution
*Biomass projections*				
Animal biomass	Projection	Fish-MIP^30^	**%**	1°
*Oceanographic*				
Temperature	Environment	NODC WOA	°C	1°
Primary production	Environment	MODIS (NASA)	gC m^−2^ yr^−1^	1°
Diatoms	Environment	Ref. ^60^	%	1°
*Fisheries productivity*				
FAO fish landings	Fisheries	FAO	t km^2^	FAO areas
Commercial fish landings	Fisheries	Ref. ^38^	kg km^−2^ yr^−1^	Ecoregion
Illegal, unreported fishery landings	Fisheries	Ref. ^38^	kg km^−2^ yr^−1^	Ecoregion
*Human stressors*				
Ocean acidity	Stressor	Ref. ^36^	–	Ecoregion
Human impact index	Stressor	Ref. ^36^	–	Ecoregion
Pollution from ship activity	Stressor	Ref. ^36^	–	Ecoregion
Hypoxia	Stressor	Ref. ^39^	%	Ecoregion
*Socioeconomic status*				
Human development index	State	United Nations	–	EEZ
Ocean health index	State	Ref. ^67^	–	EEZ
Gross domestic product	State	World bank	US$ person	EEZ
CO_2_ emissions	State	World bank	t person	EEZ
Economic adaptive capacity	State	Ref. ^68^	–	EEZ
Fishery dependency index	Pressure	Ref. ^12^	–	EEZ
Food deficit	Pressure	World bank	kcal person d^−1^	EEZ
Undernourishment	Pressure	FAO	%	EEZ
Economic vulnerability	Pressure	Ref. ^68^	–	EEZ
Food insecurity	Pressure	Ref. ^12^	–	EEZ

*Fish-MIP* Fisheries and Marine Ecosystem Model Intercomparison Project, *NODC WOA* National Oceanographic Center World Ocean Atlas, *FAO* Food and Agricultural Organization, *MODIS* moderate resolution imaging spectroradiometer, *NASA* National Aeronautics and Space Administration, *EEZ* exclusive economic zone. (–) denotes indices that are unitless.

**Fig. 3:**
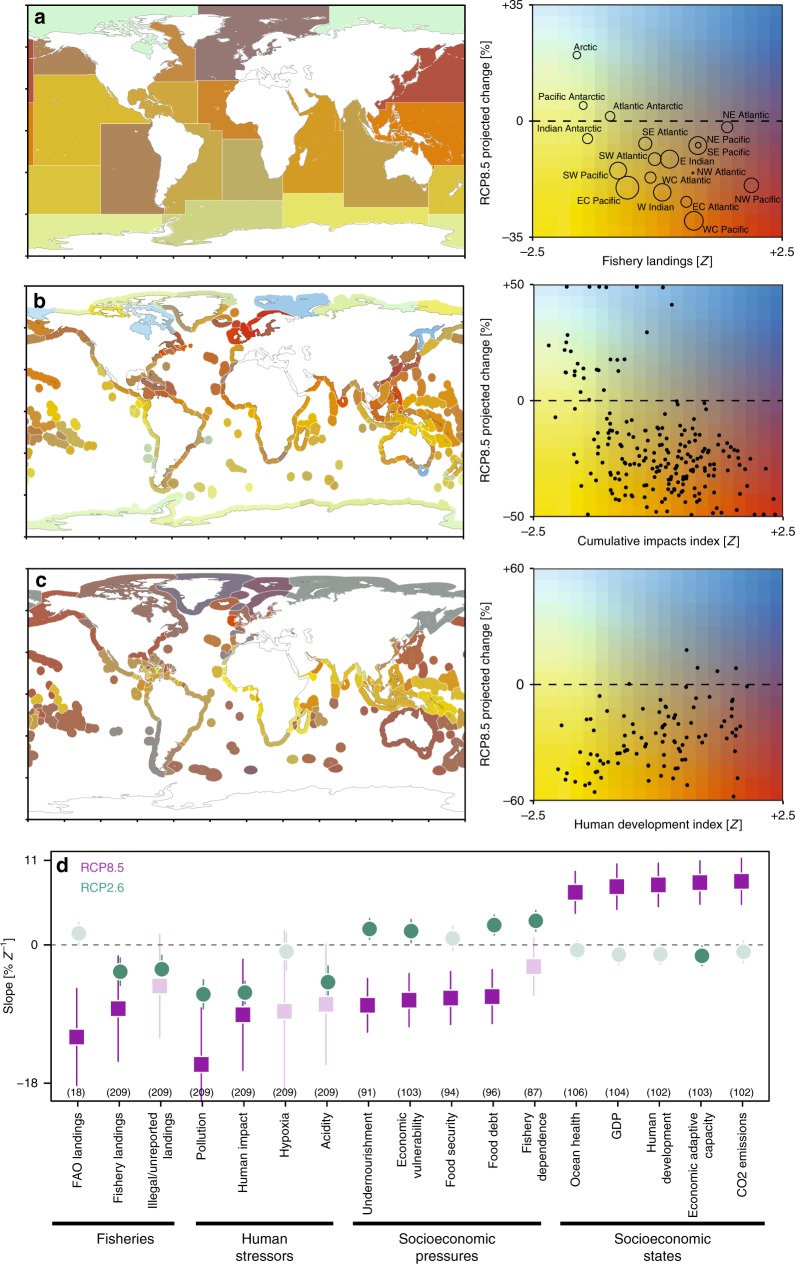
Biomass changes in relation to fisheries, stressors, and socioeconomic indicators. **a**–**c** Bivariate relationships between the projected animal biomass changes under a worst-case emission scenario (RCP8.5) and **a** total fisheries landings within 18 FAO statistical areas, **b** average cumulative human impacts within marine ecoregions, and **c** the human development index of maritime states. Bivariate maps (left panels) depict the spatial distribution of the relationships shown in the right panels. Dark and light blue depict projected biomass increase, red, orange, and yellow depict decline; horizontal line denotes no change in biomass. Symbol size in (**a**) depicts the geographic size of the FAO areas. **d** Estimated slopes from relationships between spatial gradients in 17 indicators of fisheries productivity, human stressors, and socioeconomic status (see methods for details) and projected biomass changes. Green circles are biomass changes under RCP2.6 and purple squares under RCP8.5; lines depict the 95% CIs. Darker, opaque points denote statistically significant interactions (*p* < 0.05), and lighter, semi-transparent points are non-significant. The sample sizes of the relationships are in parentheses. Negative slopes indicate stronger biomass declines in locations where indicators are largest and vice versa. All indicators in **a**–**d** have been standardized to units of variance from the mean.

Under a worst-case emission scenario (RCP8.5), consistent negative relationships emerged between projected animal biomass change and fisheries productivity: greater biomass declines were projected for areas that currently support higher fishery yields (Fig. 3a, d). This implies that fisheries yield may decline disproportionally in more productive fishing grounds. This relationship was observed using two separate sources of landings data (Fig. 3d; ref. ^38^), suggesting that it is robust across data sources and spatial scales. The Northeast Atlantic is a notable outlier to this global relationship, as it supports the second-largest fishery landings by area but is projected to experience relatively small biomass losses when averaged spatially (Fig. 3a). The apparent higher resistance of Northeast Atlantic marine ecosystems to climate effects is hypothesized to be related to elevated ocean temperatures and species diversity there, relative to the Northwest Atlantic^39,40^, which can promote stability^41^. The Arctic is another outlier, supporting virtually no fishery landings at present, but projected to experience the greatest animal biomass increases (>30%) over the next century under RCP8.5 (Fig. 3a).

Understanding future redistributions of fisheries biomass may be useful in anticipating and mitigating potential conflicts over fish and related social systems^1^. For instance, a northward shift in the distribution of Atlantic mackerel after 2007 instigated a conflict over fishing quotas between the European Union (EU), Norway, Iceland and the Faroe Islands, eroding the sustainability of the fishery^42^. The response of fisheries to projected redistributions of biomass will depend on additional factors such as profitability of fishing in potentially remote locations, which may be less accessible, the location of marine protected areas, and species-specific responses to climate change and other stressors.

Under RCP8.5, significant negative relationships were also found between the spatial distribution of biomass change and both cumulative (Fig. 3b) and individual (Fig. 3d) human stressors. This result suggests that the greatest climate-driven biomass losses will occur in locations that presently experience multiple additional human stressors, most of which are not accounted for by the MEMs used here^3^. Therefore, the biomass changes that we describe may be conservative estimates, as there will be additional impacts from fishing, bycatch, pollution, and other human impacts, which could make ecological communities more susceptible to the effects of climate change. These interactions were much weaker under the strong mitigation scenario (RCP2.6) but remained statistically significant for several indicators (green points in Fig. 3d).

Furthermore, under RCP8.5, consistent relationships were also observed between projected animal biomass changes and SES indicators (Fig. 3c, d), with more severe declines projected in regions with low SES. For example, Fig. 3c shows geographic patterns of projected biomass change and the human development index (HDI) within each EEZ (Fig. 3c, map), as well as the emergent relationship between them (Fig. 3c, right panel). The significant positive relationship between the HDI (Fig. 3c) and the mean rate of projected biomass change under RCP8.5 (*p* < 0.0001; *r*^2^ = 0.16) indicates that higher climate-driven biomass losses are projected to disproportionally occur within the EEZs of the least developed states. In addition to development status, states experiencing the greatest pressures such as high levels of undernourishment, food debt and insecurity, fishery dependency, and economic vulnerability to climate change are projected to experience the greatest losses of marine animal biomass over the coming century. These states also have the lowest ocean health scores, lowest wealth and adaptive capacity, and contribute the least to global CO_2_ emissions on a per capita (*r*^2^ = 0.13; *p* < 0.0001) and national basis (*r*^2^ = 0.1; *p* < 0.0001). The relationships between projected biomass and almost all SES indicators became weaker and often non-significant under a strong greenhouse gas mitigation scenario (RCP2.6; Fig. 3d).

Under RCP8.5, states that currently have a higher proportion of undernourishment are projected to experience the largest climate-driven reductions in animal biomass. This relationship is troubling, given that seafood accounts for 14–17% of the global animal protein consumed by humans, but with much higher reliance in small island states, where it is vital to maintaining good nutrition and health^43^. Declining animal biomass within the EEZs of states that are already experiencing poor nutrition may further exacerbate these deficiencies, particularly as these states also tend to be more dependent on fisheries, have low food security and high food debts (Fig. 3d). Changes in nutrition related to declining fisheries productivity could potentially be offset by increased agricultural production, aquaculture, or modifying food distribution systems^12^. Yet, recent studies have also highlighted the importance of seafood as a critical source of essential micronutrients that are currently lacking in the diets of up to 2 billion people^44^. These micronutrient deficiencies and their consequences are particularly severe in Asian and African countries^45,46^, many of which are projected to experience severe reductions in marine animal biomass under RCP8.5 (Fig. 2b).

### Effects of emission mitigation on animal biomass projections

**Fig. 4:**
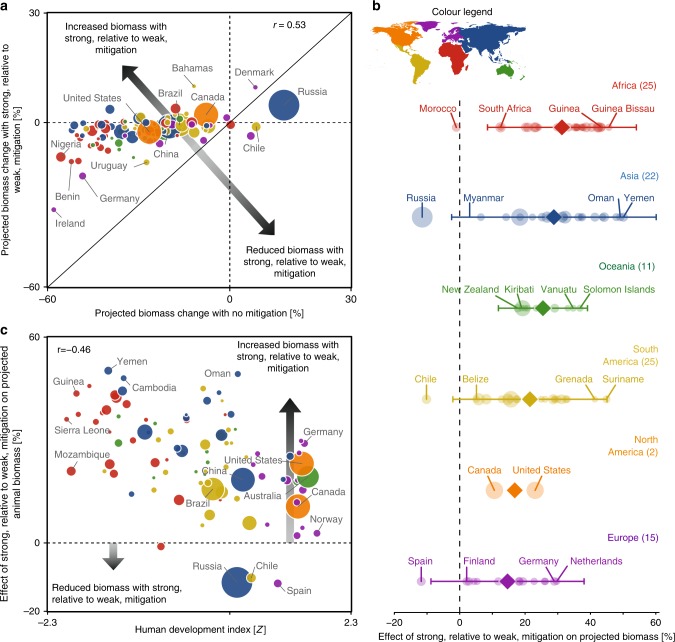
Effects of emission mitigation on projected animal biomass change. **a** Relationship between average projected animal biomass change across EEZs under a worst-case (RCP8.5) vs. strong mitigation scenario (RCP2.6). Horizontal and vertical dashed lines denote no change in projected biomass. The solid line represents a 1:1 relationship; points above this line depict EEZs in which strong (RCP2.6), relative to weak (RCP8.5), mitigation leads to greater biomass and vice versa. **b** Change in projected animal biomass resulting from strong, relative to weak mitigation within maritime EEZs (semitransparent circles) summarized within major continents (colors). The mean effect of emission mitigation on animal biomass for each continent is shown as opaque symbols with lines denoting the 95% CIs where *n* > 3. The number of EEZs is in parentheses. Points to the right of the vertical line denote biomass increases with strong emission mitigation relative to the worst-case scenario. **c** Effect of strong, relative to weak, mitigation on projected animal biomass within EEZs in relation to development status. Points above the horizontal line depict increased animal biomass with strong, relative to weak, mitigation and vice versa. For **a**–**c**, symbol size depicts the size of the EEZs and colors the continent; orange = North America, yellow = South America, purple = Europe, red = Africa, blue = Asia, and green = Oceania.

To explicitly evaluate the effect of strong emission mitigation on future animal biomass, we calculated the difference in projected biomass with the strongest mitigation scenario (RCP2.6) relative to those under a worst-case scenario (RCP8.5) within each EEZ and by continent (Fig. 4). The relationship between projected biomass under RCPs 8.5 and 2.6 was positive (*r* = 0.53) but also suggested that the effects of strong mitigation on biomass were not purely additive: some states experienced disproportionate biomass gains (Fig. 4a, above diagonal line) or losses (Fig. 4a, below diagonal line) from strong, relative to weak mitigation. Although mitigation led to increased biomass relative to worst-case emissions within the EEZs of almost all states, it resulted in declines within the EEZs of Morocco (−1%), Chile (−10%), Spain (−12%), and Russia (−12%; Fig. 4a). Relative to a worst-case scenario, the largest biomass gains from mitigation were observed for African, Asian, and South American states, including Yemen (50%), Oman (49%), Cambodia (48%), Guinea Bissau (46%), Suriname (45%), and Pakistan (44%).

On a regional basis, the largest average biomass increases due to strong, relative to weak, greenhouse gas mitigation were projected for states in Africa, Asia, Oceania, and South America, with European and North American states experiencing lower relative biomass gains (Fig. 4b). Although the average effects of mitigation were spatially variable, significant effects were apparent within Africa and Oceania. These continental-scale effects suggested that the benefits of strong relative to weak mitigation, here denoted as biomass increases, will be most experienced by states within lesser developed regions. This hypothesis was supported by examining the effect of strong relative to weak mitigation on animal biomass along gradients in the human development index (HDI; Fig. 4c). A negative correlation was found between mitigation benefits and HDI (*r* = −0.46; *p* < 0.0001), indicating substantial benefits of strong, relative to weak mitigation for the least developed states and vice versa.

## Discussion

Longitudinal models indicated that statistically significant ensemble biomass changes would occur in 40–57% of the ocean under RCPs 2.6 or 8.5, respectively (Fig. 2b, c). Our results emphasize the importance of considering statistical uncertainty for ensemble projections and propagating that uncertainty forward into subsequent analyses. The flexible and powerful longitudinal approach we use could be adopted as a general framework for estimation from ensemble projections of climate change impacts, leading to an enhanced understanding of future change and its uncertainty (see Supplementary Discussion for details).

Our analyses quantify how emissions mitigation may alter ecological changes and their consequences for states at differing levels of socio-economic development. With ongoing warming, our study suggests that the most productive fishery grounds in the ocean will experience the largest reductions in animal biomass. Should these projected trends unfold, fishery-dependent economies could be severely disrupted, and transboundary conflicts over marine resources may ensue^1^.

Our results suggest that the socioeconomic consequences of projected biomass declines under RCP8.5 will likely be disproportionately severe for coastal developing nations. Developing nations had higher cumulative human impacts, poorer ocean health, and are facing socioeconomic challenges such as lower wealth and nutrition. Developing states also tended to have a higher dependence on fisheries, rendering them more vulnerable to projected marine biomass declines due to climate change. Although developing nations have contributed the least to climate emissions, the many additional socioeconomic pressures they are facing have likely imbued them with lower adaptive capacity in the face of climate change. In addition, the majority of developing countries (e.g., India and China) have adopted more ambitious nationally determined contributions to climate mitigation (NDCs) than the average, and more than most developed states (e.g., those in Europe and North America)^47^. In a nutshell, while developing countries have contributed the least to climate change and are more aggressively curbing their emissions, they are projected to suffer the most from emission-driven impacts on their marine ecosystems and benefit the most from mitigation. To narrow these equity gaps, developed states could adopt more ambitious NDCs and prioritize the transfer of capital, technology, and adaptive capacity building to developing states.

Based on these findings, unabated climate change could seriously impede the ability of the international community to meet and maintain several of the UN SDGs, particularly those aimed at reducing hunger (SDG2), improving health, well-being (SDG3), and economic inequalities (SDG10), and avoiding adverse ecosystem effects due to climate change (SDG14). Despite these adverse outcomes, our analysis also suggests that the disproportionate climate impacts we report on developing states could be minimized through emissions mitigation. Strong mitigation would lead to increased animal biomass, relative to the worst-case emission scenario, for 96% of states, and would disproportionately benefit those in Asia, Africa, and South America (Fig. 4). Therefore, reducing emissions provides the most straightforward means of countering climate-driven biomass changes and avoiding disproportionate impacts on states that are the least well-positioned to deal with them.

## Methods

### Projected marine animal biomass data

Global projected time-series of marine animal biomass between 2006 and 2100 were obtained from the Fisheries and MEM Inter-comparison Project (Fish-MIP v1.0^3,30^), which is part of the Inter-Sectoral Impact Model Intercomparison Project (ISI-MIP). Projections were obtained from six published and validated global MEMs: APECOSM^48^, BOATS^49^, DBEM^50^, DPBM^51^, EcoOcean^52^, and Macroecological^53^. All MEMs were forced with standardized outputs from two ESMs from the Coupled Model Inter-comparison Project Phase 5 (https://esgf-node.llnl.gov/projects/cmip5): NOAA’s Geophysical Fluid Dynamics Laboratory Climate Model (GFDL-ESM2M)^54,55^ and the Institute Pierre Simon Laplace Climate Model (IPSL-CM5A-LR)^56^. These models span a broad range of the ESM projections of SST and NPP within the CMIP5 model set, GFDL-ESM2M representing the lower end of the spectrum of changes and IPSL-CM5A-LR the higher end^57^. Marine animal biomass projections (g C m^−2^) were made under RCP2.6 representing a high mitigation, low emission scenario, and RCP8.5, representing a worst-case pathway assuming a continuous increase in emissions until 2100^58^. We standardized projections to relative change to account for differences in the subsets of marine animals included in the models^3^. Although the projections used here were made without incorporating fisheries exploitation (as only a subset of models provided fished model runs), previous Fish-MIP analyses suggest that incorporating fishing does not substantially alter the projected climate-driven biomass trends^5^. Projections located within internal seas comprised 0.7% of all observations but were removed from the analyses because they are likely to be less reliable. However, retaining projections from the internal seas did not affect the results (Supplementary Table 2). A detailed overview of these models is available^3^, and the data are publicly archived^30^.

### Oceanographic data

Surface temperature (SST; °C) values over the upper 200 m were extracted from the National Oceanographic Data Center World Ocean Atlas (NODC WOA^59^) between 2005 and 2017 (Table 1). Primary production (g C m^−2^ yr^−1^) was estimated from a vertically generalized production model between 2003 and 2010. Diatom abundance estimates, expressed as the frequency of occurrence (%), were extracted from the PHYSAT project and were generated using a spectral-based method^60^.

### Projection uncertainty

The MEMs vary substantially in their underlying architecture: the processes considered, statistical assumptions, taxonomic, size class, or functional group resolutions, ecological dynamics, and species represented differ. The MEMs can be broadly categorized into those that are size-structured (BOATS, Macroecological, DBPM), species distributional (DBEM), trophodynamic (EcoOcean), or composite (APECOSM). Previous studies averaged equally (unweighted) across multiple models, yielding a single multi-model ensemble-averaged (MMEA) time-series of projected biomass from which a rate of change can be calculated; this approach is also generally used for climate-model ensemble projections^6–8^. The MMEA approach focusses on the rate of change in the averaged time-series but neglects to fully account for the variability that exists within and between the individual projections. The practical consequences of not incorporating this variability are that the MMEA approaches exclude an additional source of uncertainty, and rates of change are sensitive to outlying projections from single models, particularly when few models are available.

### Estimating global spatial patterns of standardized biomass

Spatial patterns in marine animal biomass were evaluated by first calculating mean biomass (g C m^−2^) within each grid cell and for each MEM projection over a contemporary reference period between 2006 and 2016. For each grid cell and MEM projection, we then calculated relative biomass as a proportion relative to the maximum global value. Lastly, also within each grid cell, we calculated the ensemble mean of all available standardized biomass values from each MEM projection, yielding the standardized ensemble mean biomass (%) relative to the global maximum in each cell.

### Estimating projected animal biomass change

Under each RCP scenario, each 1° grid cell could contain up to 10 biomass time series from 2006 to 2100 (not all MEMs ran with both ESM forcings). Each individual projected annual time-series was standardized to units of annual projected biomass divided by the time-series mean over the baseline reference period (2006–2016) and expressed as a percentage (i.e., 100% = no change). This data structure, in which the research units (biomass projections; *n* = 10) are tracked over time, and exhibit correlation both within and across the units is also known as longitudinal data and is exceedingly common in the health sciences, economics, and psychology studies^25–27^. Defining statistical features of longitudinal data, include (i) a hierarchical structure, whereby statistical properties must be considered both within the individual units as well as across the entire ensemble of units, and (ii) autocorrelation within the individual units. Here, we used a class of longitudinal models known as linear mixed-effects models (LMEMs)^34^. In LMEMs, the mean response is estimated as a mixture of effects: fixed effects that are assumed to be shared by all units, and random effects that vary among the individual units. The estimation of random effects enables the variation between time trends and levels of autocorrelation within them to be accounted for statistically. For each grid cell and RCP, LMEMs were used to estimate random effects that accounted for systematic differences between biomass projections from different ESM and MEM combination, and fixed effects that captured the overarching change in animal biomass and its uncertainty as

1 $$Y = X\beta + Zu + \varepsilon,$$

where *Y* are the response observations (projected biomass), *X* and *Z* are the fixed and random effects design matrices, and *β* and *u* are vectors of fixed and random effects parameters. *Y* is assumed to be Gaussian distributed. The distribution of the random effect parameters is specified as

2 $$u\sim N(0,G),$$

where *G* is the variance matrix for the random effects. The distribution for the errors is specified as

3 $$\varepsilon \sim N(0,\delta ),$$

where 0 is the mean and *δ* is the error-covariance matrix that to account for temporal autocorrelation was assumed to follow a time-dependent continuous autoregressive process. In this manner, we allowed the mean biomass (intercept) and rate of biomass change over time (slope) to vary across different biomass projections while capturing the average projected change in biomass over time and its uncertainty. This leads to highly stringent criteria for statistical significance, whereby the variation of time-dependent biomass trends is considered both within and across the biomass projection time-series. The major advantage of this approach is the ability to statistically distinguish situations where the projected changes are statistically significant from those where they are not, using a rigorous and widely accepted approach^34^. All LMEMs were estimated using the nlme package^61^ in the R statistical computing platform^62^.

### Relating projected biomass change to SES

We tested for global co-occurrence patterns between these climate-driven projections in marine animal biomass and indicators related to the current productivity of fisheries and human stressors of marine ecosystems globally. The relationship between projected biomass changes and the stressor indices may help to understand if the projected estimates of biomass change are likely to be over or under-estimations, while productivity may provide insight into how the projected changes may affect the yield and distribution of global fisheries. Indicators of fisheries productivity that we used are described in Table 1 and Supplementary Table 1 and included total fishery landings per km^2^ extracted from the Fisheries and Agricultural Organization landings database within 18 statistical areas^63^, and commercial and illegal and unreported fishery landings estimated from a range of public sources and published in from a peer-reviewed and publicly available database^38^. Indicators of human stressors included a multivariate index of human impacts on the oceans^36^, average ocean acidity^36^, ocean-based pollution from commercial and recreational ship activity^36^, and hypoxia^39^. Except for FAO fishery landings, which were available within 18 large fishery areas (Fig. 3a), all productivity and perturbation indicators were calculated within 209 marine ecoregions^37^. For each spatial unit (ecoregion or FAO area), the mean and variance of each indicator were calculated.

We also tested for co-occurrence patterns between average climate-driven projections in marine animal biomass within the EEZs of up to 106 states with coastlines >100 km and socioeconomic indicators that contain information related to the current social, economic, and ecological conditions in those states. Indicators were divided into those that contained information related to SES, where increasing values are generally interpreted to represent increasing well-being, and pressures, where increasing values represent increasing pressures acting on states. SES indicators included the per capita CO_2_ emissions, the adaptive capacity of economies to climate effects on fisheries, the HDI, per capita gross domestic product (GDP; US$), and the Ocean Health Index (OHI). SES pressures included fishery dependency, per capita food debt, the vulnerability of the economy to climate effects on fisheries between 2006 and 2100 under RCP2.6, food security, and the proportion of the population that is undernourished. Several of the indices are multivariate indicators that synthesize information related to multiple factors. For instance, the OHI assesses the health of coupled human-ocean systems by synthesizing diverse information organized into ten public goals. The HDI integrates information related to life expectancy, education, and per capita income.

To better understand the context within which the projected biomass trends occur, we analyzed linear relationships between global spatial patterns of projected biomass trends and fisheries productivity, human stressors, and socioeconomic indicators. To quantify the strength and significance of the relationships while accounting for any spatial dependence in the residuals, we fitted inverse variance-weighted generalized least squares (GLS) models^61^. The residuals from all regression models were tested using semi-variograms and Moran tests to verify that they were spatially independent. In situations where the residuals were spatially non-independent (e.g., the relationships between ecoregions), a spatial variant of the above-described GLS model was fitted to avoid any potential bias on the model inference^64^. To ensure that all regression assumptions were met, predictor variables were, if necessary, transformed to normality using Tukey’s ladder of powers^65^, which finds the power transformation which maximizes normality as assessed by Shapiro–Wilkinson tests. To enable the interactions to be compared in like units, the indices were standardized to units of variance from the mean. To avoid potentially biasing the results due to the effects of statistically uncertain biomass projections, we used only grid cells where statistically significant changes in projected biomass could be resolved (*p* < 0.05). Prior to fitting the regressions, we also removed all longitudinal trends located within the EEZs of states with short coastlines (<100 km), and in EEZs where <2 trends were available. As sensitivity checks, we also fitted the regressions using alternative model strategies or using all available trends (significant and non-significant), but the results were largely insensitive to these procedures (Supplementary Table 3). GLS models were estimated using the nlme package^61^, and Tukey transformations were estimated using the rcompanion package^66^ in the R statistical computing platform^62^.

### Reporting summary

Further information on research design is available in the Nature Research Reporting Summary linked to this article.

## Supplementary information

Supplementary Information

Reporting Summary

## Data Availability

All marine ecosystem projection data reported in this paper are described, archived and publicly available^30^. The remaining data used in this paper are available from the publicly available sources listed in Supplementary Table 1.

## Code availability

Statistical analyses were conducted using the R statistical computing platform^62^, and the code is available upon request to the corresponding author.
