# Supplementary material for: Future ocean biomass losses may widen socioeconomic equity gaps
Source: Nat Commun. 2020 May 6;11:2235. doi: 10.1038/s41467-020-15708-9 (PMC7203146; doi:10.1038/s41467-020-15708-9)
Supplement: Supplementary file 1 — Supplementary Information [file 41467_2020_15708_MOESM1_ESM.pdf]

Future ocean biomass losses may widen socioeconomic equity gaps

Daniel G. Boyce*[1,2], Heike K. Lotze[2], Derek P. Tittensor[2], David A. Carozza[3], Boris Worm[2],

[1] Ocean Frontier Institute, Dalhousie University, Halifax, Nova Scotia, Canada, B3H 4R2
[2] Department of Biology, Dalhousie University, Halifax, Nova Scotia, Canada, B3H 4R2
[3] Département de mathématiques, Université du Québec à Montreal, Montreal, Quebec, Canada, H2X 3Y7

Supplementary Information

Supplementary Methods

Longitudinal model estimates of marine animal biomass change were compared against those from ordinary linear models at local and regional scales and globally. Linear model biomass trends were estimated by firstly calculating the multi-model ensemble average biomass for each year within each grid cell, as has been undertaken previously. Following this, biomass trends were estimated from the ensemble time series within each grid cell using ordinary least-squares (OLS). Estimates of change were then averaged within 14 ocean regions that exhibit similar climatological variability and globally using inverse-variance weighting to account for trend uncertainty (longitudinal trends) or using unweighted averages (OLS).

Longitudinal model results were positively correlated with MMEA at global scales, and geographic patterns of biomass change were broadly comparable. However, longitudinal models yielded important differences in the rates of biomass change at local and regional scales. When present, differences in the rates of biomass change between the trend methods tended to be large and driven by trends identified by the longitudinal models as non-significant, emphasizing the value of considering trend certainty. Longitudinal and MMEA trends differed in the direction of biomass change in 5% of grid cells, and the total projected gain or loss of biomass differed by >100% in 2% of cells and by >5% in one-quarter (27%) of cells (Figure S1).

These differences were manifest to a greater extent when the statistical uncertainty (standard error) of the longitudinal trends was incorporated into subsequent analyses. For example, we compared averaged inverse-variance weighted longitudinal biomass trends to unweighted MMEA biomass trends across 106 exclusive economic zones, 14 large ocean regions and globally (Table S2). The averaged longitudinal, and MMEA biomass trends were highly correlated, but considering the longitudinal trend, uncertainty had a

critical effect on their interpretation (Table S2). For example, under RCP2.6, average MMEA and longitudinal biomass trends within EEZs were well correlated (r=0.99) and differed in their direction of change (+ or -) in 3% of cases. Alternatively, average MMEA trends within EEZs were less well correlated (r=0.9) to average inverse variance-weighted longitudinal trends and were different in their direction of change (+ or -) in 13% of cases. Cumulatively, these analyses suggest that the longitudinal approach we employ builds upon and refines, rather than negates, existing MMEA approaches[1–3].

Cumulatively, accounting for the statistical uncertainty of ensemble changes using longitudinal models, had a meaningful effect on the interpretation of results, as well as enabling the evaluation of the significance of observed trends. The overall trend distribution had a similar mean but a significantly larger variance (less certain) relative to MMEA, which in practice resulted in 5% of modeled trends changing from a statistically significant negative trend to an insignificant trend.

Supplementary Discussion

Despite their growing use, uncertainties in climate projections remain high, particularly at high latitudes, in nearshore areas, and inland waterways (Figures 2b, c and S2). Better constraining model performance in these situations would provide greater confidence in them, and likely increase their utility. For example, improving the ability of the ESMs and MEMs to represent dynamic nearshore processes would also be an important step in improving our understanding of future biomass changes, given the high levels of secondary production and fisheries extraction that occurs there[4]. Developing MEM projections of species or functional groups at finer spatial scales could also increase their uptake into marine management, for example, in fisheries management or climate-responsive marine spatial planning.

Incorporating the relative skill of each model into multi-model ensemble analyses, as has been attempted for climate projections (summarized in ref. [5]), could be another strategy worth considering. However, evaluating model skill requires reliable observational data, which is currently limited for marine animals at the spatial, temporal and taxonomic scales needed to fully evaluate the skill of all MEMs. Should they become available, model skill estimates could be easily implemented in the longitudinal models used here. As the number of ESMs, MEMs, and future scenarios increases, approaches such as longitudinal

modeling that can robustly combine multiple projections will become increasingly relevant, ultimately leading to improved understanding of the causes, and consequences, of climate change.

New modeling approaches, such as the longitudinal models we use, should be further developed to better quantify ensemble trends and their variability, with the aim of increasing the confidence with which they are incorporated into policy and management. The longitudinal models we use are an initial step in this direction; the models robustly estimate parameters and their uncertainty while accounting for a range of statistical complexities, including spatio-temporal dependence, linear or non-linear relationships, skewed or over-dispersed responses, different observation types (*e.g.* counts and proportions), and the use of statistical weighting[6,7].

Although the computational burden of using the multi-model longitudinal approach will increase with the number of ensemble projections, an approach that used Generalized Additive Mixed Models, possibly estimated using Integrated Nested Laplace Approximation (INLA), could be a means of increasing the computational efficiency. Freely available software packages to implement this are available in R (packages mgcv[6] and inla[8]).

Supplementary Tables

Supplementary Table 1 | Data sources. (-) denotes unitless dimensions.

Index	Category	Authority	Units	Url (www.)	Span
Biomass forecasts					
Animal biomass	Forecast	Fish-MIP[9]	**%**	dataservices.gfz-potsdam.de/pik/showshort.php?id=escidoc:2956913	2006-2100
Oceanographic					
Temperature	Environment	NODC WOA	C	nodc.noaa.gov/cgi-bin/OC5/woa18/woa18.pl	2005-2017
Primary production	Production	MODIS (NASA)	g C m^{-2} yr^{-1}	oceancolor.gsfc.nasa.gov/	2003-2010
Diatoms	Environment	Ref. [10]	%	www.sciencedirect.com/science/article/pii/S0967063705001536	1998-2010
Global productivity and stress					
FAO fish landings	Fisheries	FAO	t km^2	fao.org/fishery/collection/global-production/en	1990-2010
Fish landings	Fisheries	Ref. [11]	kg km^{-2} yr^{-1}	dx.doi.org/10.4226/77/58293083b0515	1950-2015
Illegal, unreported fishery landings	Fisheries	Ref. [11]	kg km^{-2} yr^{-1}	dx.doi.org/10.4226/77/58293083b0515	1950-2015
Human Stressors					
Ocean acidity	Stressor	Ref. [12]	-	nceas.ucsb.edu/globalmarine	1870-2009
Human impact index	Stressor	Ref. [12]	-	nceas.ucsb.edu/globalmarine	2008
Pollution from ship activity	Stressor	Ref. [12]	-	nceas.ucsb.edu/globalmarine	2008
Hypoxia	Stressor	Ref. [13]	%	nodc.noaa.gov/cgi-bin/OC5/woa18/woa18oxnu.pl?parameter=o	1990-200
Socioeconomic states					
Human development index	State	United Nations	-	hdr.undp.org/en/data#	1990-2017
Ocean health index	State	Ref. [14]	-	oceanhealthindex.org/	2012
Gross domestic product	State	World bank	US$ person	data.worldbank.org/indicator/ny.gdp.mktp.cd	1990-2016
CO2 emissions	State	World bank	t person	data.worldbank.org/indicator/EN.ATM.CO2E.PC?view=chart	1990-2018
Economic adaptive capacity	State	Ref. [15]	-	journals.plos.org/plosone/article?id=10.1371/journal.pone.0179632	2019
Fishery dependency index	Pressure	Ref. [2]	-	nature.com/articles/s41559-017-0258-8	2017
Food deficit	Pressure	World bank	kcal person d^{-1}	ourworldindata.org/hunger-and-undernourishment#depth-of-the-food-deficit	1999-2001
Human impact index	Pressure	FAO	-	nceas.ucsb.edu/globalmarine	2015
Undernourishment	Pressure	Ref. [15]	%	ourworldindata.org/hunger-and-undernourishment	2014-2016
Economic vulnerability	Pressure	Ref. [2]	-	journals.plos.org/plosone/article?id=10.1371/journal.pone.0179632	2019
Food security	Pressure	Ref. [2]	-	nature.com/articles/s41559-017-0258-8	2017

Notes: NODC WOA: National Oceanographic Center World Ocean Atlas; FAO: Food and Agricultural Organization; MODIS: Moderate Resolution Imaging Spectroradiometer; NASA: National Aeronautics and Space Administration; SEDAC: Socioeconomic Data and Applications Center; CIA: Central Intelligence Agency.

Supplementary Table 2 | Global patterns of multi-model animal biomass change estimated using all available data, or removing estimates within internal waterways (*e.g.* Figure 2).

		RCP2.6				RCP8.5			
	**n**	**Significant (%)**	**Increasing (%)**	**Declining (%)**	**Median**	**Significant (%)**	**Increasing (%)**	**Declining (%)**	**Median**
**Full dataset**	82562	39.9	32.1	67.9	-0.01	56.7	16.3	83.7	-0.11
**No internal seas**	81956	39.9	32.4	67.6	-0.01	56.7	16.3	83.7	-0.11

Supplementary Table 3 | Regional and globally averaged ensemble estimates of biomass change estimated from traditional multi-model ensemble averaged-ordinary least-squares approach (MMA), and from longitudinal models (LME).

	RCP 2.6				RCP 8.5			
**Region**	**n**	**MMA trend (%)**	**LME trend (%)**	**Δ**	**n**	**MMEA trend (%)**	**LME trend (%)**	**Δ**
**Arctic**	6242	29.7	2.6	-27.1	6277	265.7	20.7	-245
**Black**	46	53.6	33.6	-20	46	108.4	70.8	-37.6
**E Atlantic**	2121	-1.2	-2.4	-1.2	2169	-26.9	-25	1.9
**E Pacific**	6167	-6.7	-5.9	0.8	6211	-28.2	-25.4	2.8
**Med.**	221	10.5	5.3	-5.2	226	-26.7	-27.3	-0.6
**N Atlantic**	2675	5.2	1	-4.2	2742	-21	-21.1	-0.1
**N Indian**	902	-2.8	-2.9	-0.1	897	-39.4	-37.7	1.7
**N Pacific**	4099	4.4	1	-3.4	4143	-14.9	-21	-6.1
**S Atlantic**	3030	22.5	1.5	-21	3078	3723.4	-5.1	-3728.5
**S Indian**	5768	4.2	-2.1	-6.3	5904	34.2	-13.3	-47.5
**S Pacific**	5270	5.2	0.4	-4.8	5387	-0.4	-12.9	-12.5
**Southern**	3879	-0.4	-0.5	-0.1	3970	12.4	5.4	-7
**Global**	40420	7.3	-1.4	-8.7	41050	316.4	-12.8	-329.2

Notes: Δ=difference between MMA and LME trend estimates; MMA=multi-model average; LME=linear mixed-effects.

Supplementary Table 4 | Relationships between indicators of fisheries production, human stressors, and SES status and forecasted biomass changes estimated using different univariate regression model approaches. Bold denotes statistically significant relationships ($p<0.05$).

			Weighted spatial GLS		Weighted GLS		Weighted robust model	
**RCP**	**Category**	**Index**	**Trend**	**P-value**	**Trend**	**P-value**	**Trend**	**P-value**
2.6	Fisheries	Fishery landings	**-2.73**	**0**	**-5.2**	**0**	-0.5	0.2
2.6	Fisheries	Illegal/unreported landings	**-2.4**	**0.01**	**-4.49**	**0**	-0.43	0.26
2.6	SES	CO2 emissions	-0.72	0.32	-0.91	0.21	0.56	0.17
2.6	SES	Economic adaptive capacity	**-1.45**	**0.03**	**-1.42**	**0.03**	0.03	0.94
2.6	SES	Economic vulnerability (RCP2.6)	**1.65**	**0.03**	**1.8**	**0.02**	-0.55	0.29
2.6	SES	Fishery dependence	**2.99**	**0**	**3.13**	**0**	-0.32	0.44
2.6	SES	Food debt	**2.51**	**0**	**2.58**	**0**	-0.39	0.32
2.6	SES	Food security	0.63	0.44	0.84	0.31	**-1.22**	**0**
2.6	SES	GDP	-1.15	0.1	-1.27	0.07	0.41	0.28
2.6	SES	Human development	-1.11	0.1	-1.22	0.08	0.55	0.14
2.6	SES	Ocean health	-0.54	0.4	-0.7	0.28	0.63	0.1
2.6	SES	Undernourishment	**1.96**	**0**	**2.07**	**0**	-0.53	0.23
2.6	Stressor	Acidity	**-5.33**	**0**	**-6.21**	**0**	-0.84	0.06
2.6	Stressor	Human impact	**-6.97**	**0**	**-7.3**	**0**	**-1.1**	**0.01**
2.6	Stressor	Hypoxia	0.33	0.78	**-3.88**	**0**	0.25	0.4
2.6	Stressor	Pollution	**-5.51**	**0**	**-6.25**	**0**	**-0.87**	**0.05**
8.5	Fisheries	Fishery landings	**-7.8**	**0**	**-15.25**	**0**	**-3.47**	**0**
8.5	Fisheries	Illegal/unreported landings	**-6.9**	**0**	**-12.46**	**0.01**	**-3.82**	**0**
8.5	SES	CO2 emissions	**8.27**	**0**	**8.27**	**0**	**5.41**	**0**
8.5	SES	Economic adaptive capacity	**8.11**	**0**	**8.11**	**0**	3.3	0.07
8.5	SES	Economic vulnerability (RCP2.6)	**-4.29**	**0.03**	**-7.19**	**0**	**-3.99**	**0.01**
8.5	SES	Fishery dependence	-0.47	0.82	-2.83	0.14	-2.65	0.09
8.5	SES	Food debt	-4.14	0.03	**-6.73**	**0**	-4.03	0.02
8.5	SES	Food security	**-6.91**	**0**	**-6.91**	**0**	**-5.7**	**0**
8.5	SES	GDP	**7.58**	**0**	**7.58**	**0**	**5.41**	**0**
8.5	SES	Human development	**7.81**	**0**	**7.81**	**0**	**6.01**	**0**
8.5	SES	Ocean health	**6.31**	**0**	**6.84**	**0**	**6.33**	**0**
8.5	SES	Undernourishment	**-7.86**	**0**	**-7.86**	**0**	**-5.15**	**0**
8.5	Stressor	Acidity	**-9**	**0.01**	**-19.49**	**0**	**-7.14**	**0**
8.5	Stressor	Human impact	**-7.14**	**0**	**-22.91**	**0**	**-4.15**	**0**
8.5	Stressor	Hypoxia	-5.87	0.06	**-20.35**	**0**	-1.11	0.2
8.5	Stressor	Pollution	**-11.42**	**0**	**-26.23**	**0**	**-5.47**	**0**

Supplementary Figures

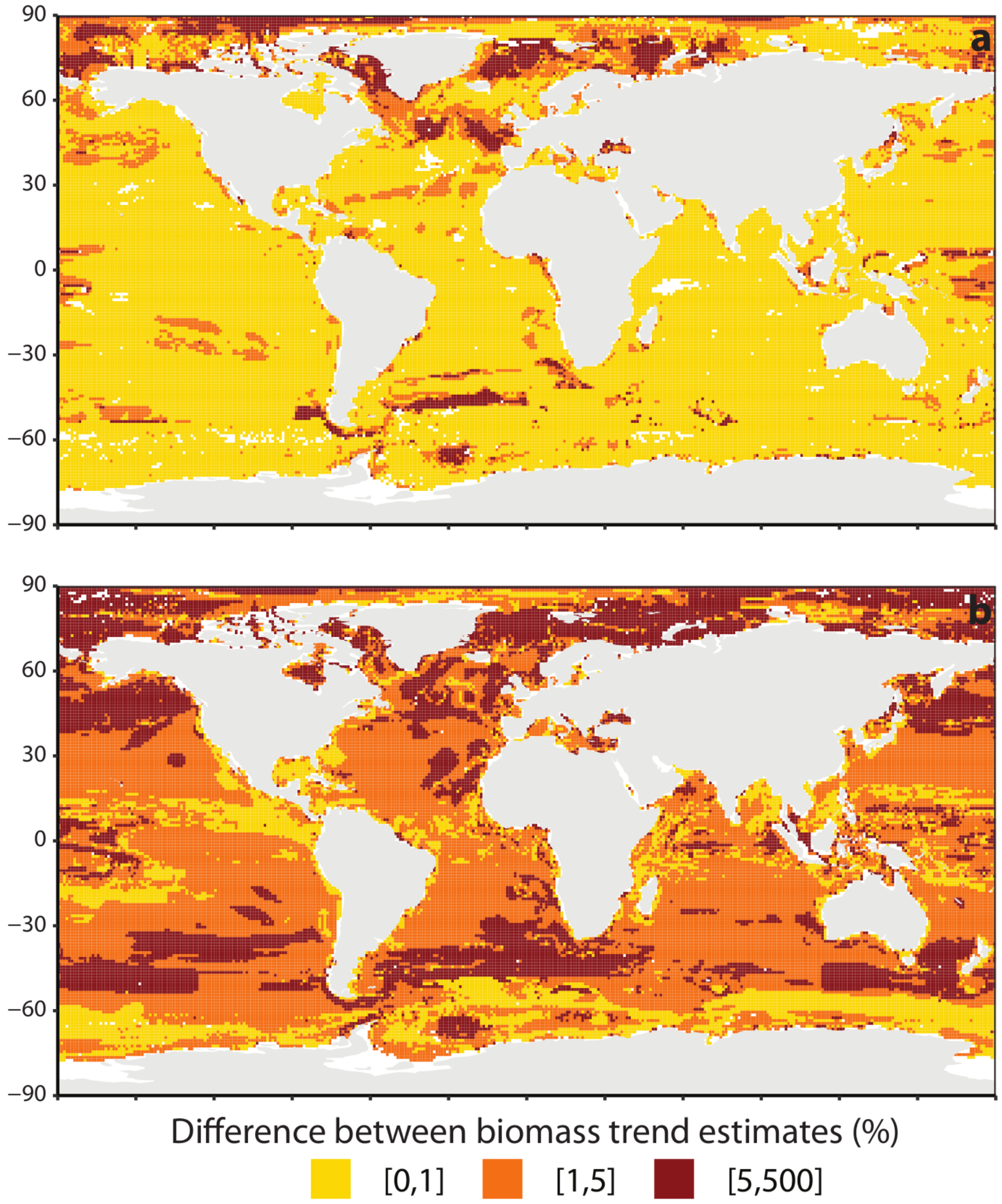

Supplementary Figure 1 | Difference in absolute biomass change between 2006 and 2098 when estimated using longitudinal models compared to ordinary least squares model fitted to multi-model averaged time-series. Colours are the difference in estimated biomass gains/losses between the two approaches binned into low (0-0.99%; yellow), medium (1-4.99; orange), and high (>5; red) difference categories. Differences were calculated under (a) RCP2.6 and (b) RCP8.5.

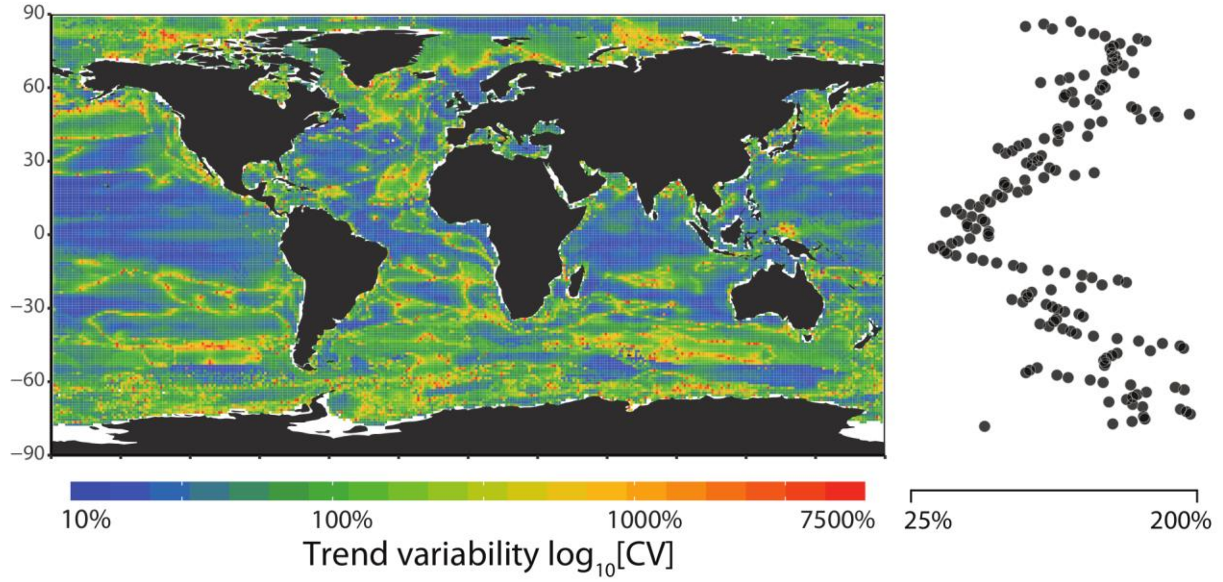

Supplementary Figure 2 | Global patterns of standardized uncertainty in projected in marine animal biomass changes. Standardized uncertainty of forecasted biomass time trends, as the coefficient of longitudinal model trend variation (σ_t/β_t) estimated globally (left) and along latitude (right). Blue depicts the lowest trend variation and red the highest; white depicts areas where the longitudinal models did not converge, or data was insufficient.

Supplementary References

1. Lotze, H. K. *et al.* Ensemble projections of global ocean animal biomass with climate change. *Proc. Natl. Acad. Sci.* 1–6 (2019). doi:doi.org/10.1073/pnas.1900194116
2. Blanchard, J. L. *et al.* Linked sustainability challenges and trade-offs among fisheries, aquaculture and agriculture. *Nat. Ecol. Evol.* **1**, 1240–1249 (2017).
3. Bryndum-Buchholz, A. *et al.* 21 St Century Climate Change Impacts on Marine Animal Biomass and Ecosystem Structure Across Ocean Basins. *Glob. Chang. Biol.* **25**, 459–472 (2018).
4. Holt, J. *et al.* Prospects for improving the representation of coastal and shelf seas in global ocean models. *Geosci. Model Dev.* **10**, 499–523 (2017).
5. Eyring, V. *et al.* Taking climate model evaluation to the next level. *Nat. Clim. Chang.* **9**, 102–110 (2019).
6. Wood, S. N. *Generalized additive models: an introduction with R*. *Biometrics* **62**, (Chapman & Hall/CRC, 2006).
7. Hastie, T. & Tibshirani, R. Generalized additive models. *Stat. Sci.* **1**, 297–318 (1986).
8. Rue, H., Martino, S. & Chopin, N. Approximate Bayesian inference for latent Gaussian models using integrated nested Laplace approximations (with discussions). *J. R. Stat. Soc. Ser. B* **71**, 392 (2009).
9. Tittensor, D. P. *et al.* ISIMIP2a Simulation Data from Fisheries & Marine Ecosystems (Fish-MIP; Global) Sector. Potsdam Institute for Climate Impact Research.GFZ Data Services. http://doi.org/10.5880/PIK.2018.005. Deposited 31 January 2018. (2018).
10. Alvain, S. *et al.* Remote sensing of phytoplankton groups in case 1 waters from global SeaWiFS imagery. *Deep Sea Res. Part I Oceanogr. Res. Pap.* **52**, 1989–2004 (2005).
11. Watson, R. A. A database of global marine commercial, small-scale, illegal and unreported fisheries catch 1950-2014. *Sci. Data* **4**, 1–9 (2017).
12. Halpern, B. S. *et al.* A global map of human impact on marine ecosystems. *Science* **319**, 948–952 (2008).
13. Boyce, D. G., Frank, K. T., Worm, B. & Leggett, W. C. Spatial patterns and predictors of trophic

control across marine ecosystems. *Ecol. Lett.* **18**, 1001–1011 (2015).

14. Halpern, B. S. *et al.* An index to assess the health and benefits of the global ocean. *Nature* **488**, 615–620 (2012).

15. Blasiak, R. *et al.* Climate change and marine fisheries: Least developed countries top global index of vulnerability. *PLoS One* **12**, 1–15 (2017).
