# Supplementary material for: Future ocean biomass losses may widen socioeconomic equity gaps
Source: Nat Commun. 2020 May 6;11:2235. doi: 10.1038/s41467-020-15708-9 (PMC7203146; doi:10.1038/s41467-020-15708-9)
Supplement: Supplementary file 2 — Reporting Summary [file 41467_2020_15708_MOESM2_ESM.pdf]

nature research

Corresponding author(s): Daniel G. Boyce
Last updated by author(s): Mar 4, 2020

Reporting Summary

Nature Research wishes to improve the reproducibility of the work that we publish. This form provides structure for consistency and transparency in reporting. For further information on Nature Research policies, seeAuthors & Referees and theEditorial Policy Checklist .

Statistics

For all statistical analyses, confirm that the following items are present in the figure legend, table legend, main text, or Methods section.

n/a	Confirmed	
☐	☒	The exact sample size (*n*) for each experimental group/condition, given as a discrete number and unit of measurement
☒	☐	A statement on whether measurements were taken from distinct samples or whether the same sample was measured repeatedly
☐	☒	The statistical test(s) used AND whether they are one- or two-sided<br>*Only common tests should be described solely by name; describe more complex techniques in the Methods section.*
☐	☒	A description of all covariates tested
☐	☒	A description of any assumptions or corrections, such as tests of normality and adjustment for multiple comparisons
☐	☒	A full description of the statistical parameters including central tendency (e.g. means) or other basic estimates (e.g. regression coefficient) AND variation (e.g. standard deviation) or associated estimates of uncertainty (e.g. confidence intervals)
☐	☒	For null hypothesis testing, the test statistic (e.g. *F*, *t*, *r*) with confidence intervals, effect sizes, degrees of freedom and *P* value noted<br>*Give P values as exact values whenever suitable.*
☒	☐	For Bayesian analysis, information on the choice of priors and Markov chain Monte Carlo settings
☒	☐	For hierarchical and complex designs, identification of the appropriate level for tests and full reporting of outcomes
☐	☒	Estimates of effect sizes (e.g. Cohen's *d*, Pearson's *r*), indicating how they were calculated

Our web collection on statistics for biologists contains articles on many of the points above.

Software and code

Policy information about availability of computer code

Data collection	No software was used.
Data analysis	As mentioned in our ms, we used the freely available R statistical computing platform (ver. 3.6.1) and the associated packages nlme, rcompanion, and robust to conduct the statistical analyses. MatLab was used to extract and format the data sets prior to analyses.

For manuscripts utilizing custom algorithms or software that are central to the research but not yet described in published literature, software must be made available to editors/reviewers. We strongly encourage code deposition in a community repository (e.g. GitHub). See the Nature Research guidelines for submitting code & software for further information.

Data

Policy information about availability of data

All manuscripts must include a data availability statement. This statement should provide the following information, where applicable:

- Accession codes, unique identifiers, or web links for publicly available datasets
- A list of figures that have associated raw data
- A description of any restrictions on data availability

All data used in this paper are publicly available referenced within the ms. Forecast data are publicly available at http://dataservices.gfz-potsdam.de/pik/showshort.php?id=escidoc:2956913. The remaining data sets can be accessed through the sources listed in Table S1.

Field-specific reporting

Please select the one below that is the best fit for your research. If you are not sure, read the appropriate sections before making your selection.

Ecological, evolutionary & environmental sciences study design

All studies must disclose on these points even when the disclosure is negative.

Study description	We used linear mixed models to estimate time trends in animal biomass between 2006-2098 from global ecosystem model ensemble forecasts (n=10). Trends were estimated for each of up to 64,800 1x1 degree grid cells globally under two contrasting emission scenarios. The relationship between ensemble trends and indicators of fisheries productivity (n=3), human stresors (n=4), and socioeconomic factors (n=10) was estimated using inverse-variance weighted spatial generalized least squares models.
Research sample	All forecasted data reported in this paper are publicly available at http://dataservices.gfz-potsdam.de/pik/showshort.php?id=escidoc:2956913. Remaining data used in this paper are available from the publicly available sources listed in Table S1.
Sampling strategy	The sample sizes were standardized across the forecasts used and determined by the Fish-MIP protocols.
Data collection	All forecasted data reported in this paper are published and described and are archived and publicly available at http://dataservices.gfz-potsdam.de/pik/showshort.php?id=escidoc:2956913.
Timing and spatial scale	This study used annual forecasts of marine animal biomass available between 2006 and 2100 obtained through the Fish-MIP program. These data are publicly available at http://dataservices.gfz-potsdam.de/pik/showshort.php?id=escidoc:2956913
Data exclusions	We did not exclude data.
Reproducibility	To ensure reproducibility, all datasets are publicly available through the sources listed in our ms and the code used to analyze the data will be released through Github should our paper be published.
Randomization	Not relevant to our study
Blinding	Not relevant to our study.

Did the study involve field work? ☐ Yes ☒ No

Reporting for specific materials, systems and methods

We require information from authors about some types of materials, experimental systems and methods used in many studies. Here, indicate whether each material, system or method listed is relevant to your study. If you are not sure if a list item applies to your research, read the appropriate section before selecting a response.

Materials & experimental systems

n/a	Involved in the study
☒	☐ Antibodies
☒	☐ Eukaryotic cell lines
☒	☐ Palaeontology
☒	☐ Animals and other organisms
☒	☐ Human research participants
☒	☐ Clinical data

Methods

n/a	Involved in the study
☒	☐ ChIP-seq
☒	☐ Flow cytometry
☒	☐ MRI-based neuroimaging
